# 

**Published:** 1823-08

**Authors:** 


### MONTHLY CATALOGUE OF MEDICAL BOOKS.

*** *It having been hinted to us that gentlemen, sending copies of their Works, prefer having their titles given at length, it is our intention, in future, to comply with this suggestion: but it is to be observed, that no books can be entered on this list except those sent to us for the purpose;—in the lists hitherto transmitted, we find that the names of works have frequently been given as published, which have not appeared for weeks, or even months after.*

A Series of Elementary Lectures on the Veterinary Art, wherein the Anatomy, Physiology, and Pathology of the Horse are essayed on the general Principles of Medical Science. By Veterinary-Surgeon PERCIVAL, of the Royal Regiment of Artillery. 8vo. pp. xxxvi. 380.—London, 1823.

Observations in Surgery. By HENRY EARLE, F.R.S. Assistant Surgeon to St. Bartholomew's Hospital, and Surgeon to the Foundling.—pp. 229. Underwood, London, 1823.

---

### NOTICES TO CORRESPONDENTS.

Mr. J.'s *Paper on Diseases of the Liver is not calculated for publication: we must therefore decline inserting it.*

Mr. M. *will excuse our not noticing the criticism contained in the work he alludes to.*

*We beg to thank* Mr. HUTCHINSON *for his polite letter: his wishes shall be attended to.*

*In our Number for June, under the head of* "Intelligence," *a case of Boulimia, by* Dr. CRANE, *was inserted from* HUFELAND's *Journal: we soon after discovered that this case had been published two years previously. Our apology must be, that we were not then in the habit of regularly perusing the monthly Journals, and were consequently ignorant of the fact.*

